# Geographic availability to optometry services across Canada: mapping distribution, need and self-reported use

**DOI:** 10.1186/s12913-020-05499-6

**Published:** 2020-07-10

**Authors:** Tayyab Shah, Stephan Milosavljevic, Brenna Bath

**Affiliations:** 1 School of Rehabilitation Science, University of Saskatchewan, Suite 3400, 3rd Floor, 104 Clinic Pl, Saskatoon, Saskatchewan S7N 2Z4 Canada; 2 School of Geography, Earth Science, and Environment, University of the South Pacific, Suva, Fiji; 3 School of Rehabilitation Science, University of Saskatchewan, Rm 3410, Health Sciences Building, 104 Clinic Place PO Box 23, Saskatoon, Saskatchewan S7N 2Z4 Canada; 4 School of Rehabilitation Science and Canadian Centre for Health and Safety in Agriculture (CCHSA), University of Saskatchewan, Rm 1340 - E wing - Health Sciences Building, 104 Clinic Place PO Box 23, Saskatoon, Saskatchewan S7N 2Z4 Canada

**Keywords:** Optometry services, GIS, Access to vision care, Spatial patterns, Canadian health regions, Primary health care

## Abstract

**Background:**

This research investigates the distribution of optometrists in Canada relative to population health needs and self-reported use of vision services.

**Methods:**

Optometrist locations were gathered from provincial regulatory bodies. Optometrist-to-population ratios (i.e. the number of providers per 10,000 people at the health region level) were then calculated. Utilization of vision care services was extracted from the Canadian Community Health Survey (CCHS) 2013–2014 question regarding self-reported contacts with optometrists or ophthalmologists. Data from the 2016 Statistics Canada census were used to create three population ‘need’ subgroups (65 years and over; low-income; and people aged 15 and over with less than a high school diploma). Cross-classification mapping compared optometrist distribution to self-reported use of vision care services in relation to need. Each variable was converted into three classes (i.e., low, moderate, and high) using a standard deviation (SD) classification scheme where ±0.5SD from the mean was considered as a cut-off. Three classes: low (< − 0.5SD), moderate (− 0.5 to 0.5SD), and high (> 0.5SD) were used for demonstrating distribution of each variable across health regions.

**Results:**

A total of 5959 optometrists across ten Canadian provinces were included in this analysis. The nationwide distribution of optometrists is variable across Canada; they are predominantly concentrated in urban areas. The national mean ratio of optometrists was 1.70 optometrists per 10,000 people (range = 0.13 to 2.92). Out of 109 health regions (HRs), 26 were classified as low ratios, 51 HRs were classified as moderate ratios, and 32 HRs were high ratios. Thirty-five HRs were classified as low utilization, 39 HRs were classified as moderate, and 32 HRs as high utilization. HRs with a low optometrist ratio relative to eye care utilization and a high proportion of key sociodemographic characteristics (e.g. older age, low income) are located throughout Canada and identified with maps indicating areas of likely greater need for optometry services.

**Conclusion:**

This research provides a nationwide overview of vision care provided by optometrists identifying gaps in geographic availability relative to “supply” and “need” factors. This examination of variation in accessibility to optometric services will be useful to inform workforce planning and policies.

## Background

Primary health care (PHC) in Canada includes a variety of services from a range of health professionals providing comprehensive care and coordination with other levels of care [1–4]. Access to PHC services is a considerable health delivery concern across Canada with important health policy implications. Some communities, particularly in rural and remote areas, do not have the same access to a range of primary health care professionals [5, 6]. Such differences in access to health services have negative consequences for best meeting population health needs. Geographic access to PHC services involves investigating the distribution of these services in relation to population health needs [7–9]. The increasing interest in geographic access to PHC services in Canada has focused predominantly on physicians, and dentists [10–16]. In relation to vision care services, the few studies in Canada to date have focused on either ophthalmologists only [17, 18] or combined distribution of ophthalmologists and optometrists [19]. Optometrists are identified as “independent primary health care providers and represent the front line of vision health” (CAO website) and practice in a diverse range of settings across Canada including private practice, community health centres, and hospitals [20]. In Canada, no referral is required to access optometrist while ophthalmologists usually require a referral from family physicians or optometrists. Research investigating vision care provider use (i.e. optometrist or ophthalmologist) based on a self-reported national survey in Canada, found that populations having high risk of vision loss may lack access to eye care services [21], socioeconomic characteristics may be barriers to eye service utilization among certain subgroups [22], and those without additional health insurance have reduced use and access to eye care services [23]. However, having additional health care insurance does not necessarily result in equitable access to eye care services. Residents of US with low densities of eye care professionals, for example, have reduced likelihood of vision service access, even among those with insurance [24]. Further, rural and remote residents face additional challenges due to longer travel distances to receive vision care [25].

To date, there has been little work in Canada that has focused solely on investigating the distribution of optometry services relative to potential need and use. The aim of this research was to explore the distribution of optometrists in relation to population health needs and self-reported use of vision care services across Canada in order to identify potential gaps in geographic access relative to “supply” of and “need” for such factors. Specifically, this research: 1) identifies variation and poorly-served areas (i.e. health regions) to optometry services across Canada; 2) analyzes and maps the self-reported use of vision care services in relation to optometrist distribution; and 3) maps the patterns of spatial distribution of optometrists in relation to census-based socio-demographic characteristics (e.g. age, income, education).

## Methods

**Fig. 1:**
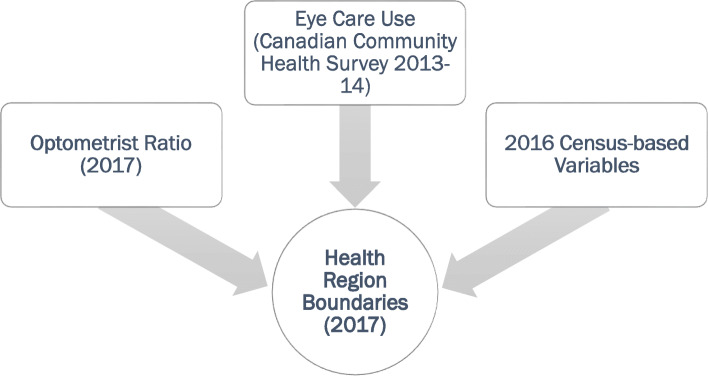
Data sources

This research is based on the number of optometrists per population (i.e. optometrist distribution ratio), eye services utilization (including optometrists and ophthalmologists), and population subgroups that may have higher health care needs (i.e. sociodemographic characteristics such as seniors population, low-income measures, and less education) (see Fig. 1) [22, 26–28]. The primary practice locations of optometrists in Canada were gathered from the provincial regulatory bodies. The Canadian Association of Optometrists (CAO) gathered primary practice information of optometrists for 2017 (i.e. six-digit postal codes) from seven provinces (British Columbia, Manitoba, New Brunswick, Ontario, Prince Edward Island, Quebec, Saskatchewan) whereas data from the remaining three provinces (i.e. Alberta, Newfoundland and Labrador, Nova Scotia) was downloaded directly from each of the provincial regulatory college’s website. CAO was unable to provide optometrist information for three Territories due to unavailability of any licensing bodies to provide data. A set of geographic coordinates for primary practice locations were generated using postal code geocoding and Google Maps. Next, in order to aggregate data in various geographic scales, supporting attributes from other layers such as health region boundaries, census subdivision (CSD) geographic units were assigned to each location.

Geographic proximity of optometry services was measured in terms of the number of optometrists per 10,000 population at health region levels (i.e. optometrist distribution ratio). The number of optometrists extracted from the provincial regulatory bodies (December 2017 to July 2018) from either the 2017 or 2018 registration year combined with Census derived population figures were used for estimating optometrist ratios at a health region level. Information about utilization of eye care services (i.e. combination of optometrist or ophthalmologist) is based on the CCHS 2013–2014 that was accessed via Ontario Data Documentation, Extraction Service and Infrastructure (odesi) web-based data exploration, extraction, and analysis tool (https://odesi.ca/). The CCHS is a cross-sectional, nationwide, and self-reported household survey that was collected from persons aged 12 and over living in Canadian health regions except those living on a reserve or as fulltime member of the Canadian Forces [29]. To get a fair sample distribution to the health regions and the provinces, the CCHS survey adopted a multi-stage sample allocation strategy including each provinces’ sample is allocated among its health regions as per their size of the population [29]. We used the following question to derive the information about utilization of eye care services at health regions: “CHP_Q06: [Not counting when you were an overnight patient, in the past 12 months/In the past 12 months], have you seen, or talked to: an eye specialist, such as an ophthalmologist or optometrist (about your physical, emotional or mental health)?” Unfortunately, the wording of the CHP question related to vision care services does not distinguish between optometrist or ophthalmologist use. Comparative analyses of ratio and utilization variables in association with population subgroups that usually have much higher health care needs was performed. We focused on the following three population subgroups with potentially higher needs: seniors (age 65 years and over), low-income, and lower educational attainment. Information about these three variables were extracted from 2016 Census and downloaded from the Statistics Canada website. The 2016 dissemination area (DA) census data were used to prepare the following HR level variables: population 65 years and over, low-income measures (after tax), and the population aged 15 and over with less than a high school diploma. These variables were expressed as percentages. Low-income measures is one of three measures of low income in Canada that calculates relative measures of low income based on the national income distribution where an adjustment of 50% median household income is set as a threshold [30, 31]. In this study, data were gathered from multiple sources at various geographic levels such as optometrist use at health regions, population subgroups at DA, and optometrist work location at locational scale, however, health regions are used as the unit of analysis.

Geospatial mapping methods that were used to analyze the patterns of optometrists per 10,000 population (i.e. ratio), self-reported eye care services utilization, and population subgroups can be divided into three ways. First, optometrist practice locations were associated with the different urban-rural classifications where we used statistical area classification to categorize census subdivisions (municipalities) into metropolitan and metropolitan influence zones (MIZs) [32]. The MIZ classifies the CSDs outside census metropolitan areas (CMAs) and census agglomerations (CA) into four categories according to the degree of influence (strong, moderate, weak, or no influence) that the CMAs or CAs have on them [32]. These categories are based on the proportion of employed residents in a given CSD that commute to work in a CMA or CA (i.e. strong > 30%, moderate 5–30%, weak< 5%, no influence 0 residents) [32]. Second, optometrist ratios estimated at health regions levels were mapped. This was done after converting ratio values into five categories where a standard deviation (SD) classification approach was used (± 0.5 SD from the mean value were used as a cut-off for demonstrating distribution of optometrists across health regions) [33, 34]. Third, a cross-classification technique was utilized to map the patterns of spatial distribution of optometrists in relation to self-reported use of vision care services and population subgroups. This was performed after separating each variable into three classes (i.e., low, moderate, and high). For this, a standard deviation classification scheme was followed where a ± 0.5 SD from the mean value was used as a cut-off for demonstrating distribution of each variable across health regions. For example, in case of optometrist ratio, the first two categories (< − 1.5 SD; − 1.5 to - 0.50 SD) indicate poor distribution of optometrists (i.e., lower category), the third category (− 0.5 to 0.5 SD) moderate, and the last two (0.5 to 1.5 SD, > 1.5 SD) indicate higher geographical availability of optometry services.

The following software were used for mapping and data analysis (spatial and nonspatial): ArcGIS Map, SPSS, and Microsoft Excel. A thematic mapping tool available in ArcGIS software (ArcGIS Desktop version 10.5, ESRI, Redlands, CA) was used to prepare a set of maps. Supporting datasets required for mapping were accessed by the research team through the Geographical Information System (GIS) Library Services at the University of Saskatchewan [35]. These datasets included a digital geographic boundary file for health regions, demographic data, digital geographic file of the 2016 Canadian Census at various geographic scales, and CanMap Postal Code Suite for geocoding purposes.

## Results

**Fig. 2:**
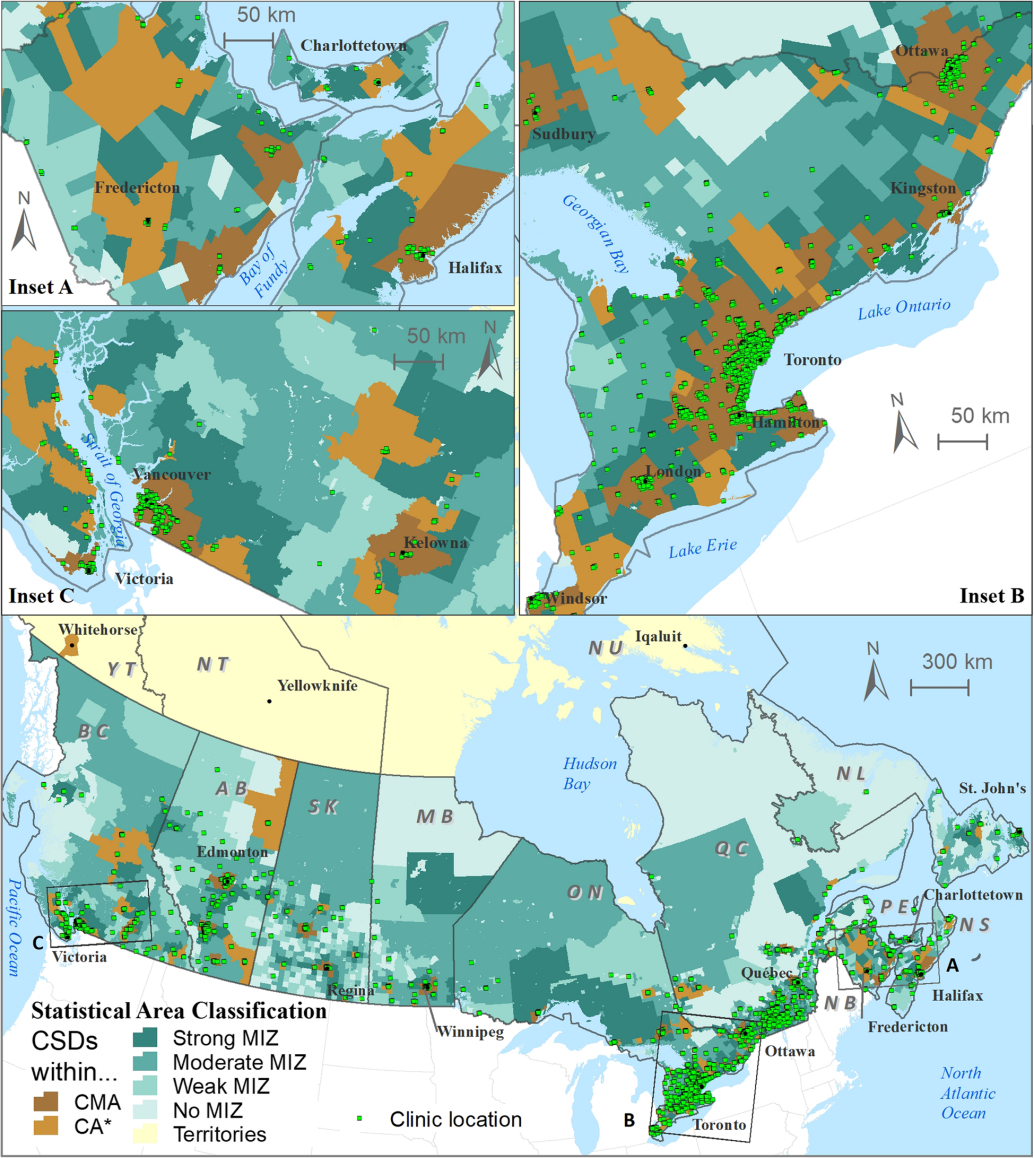
Distribution of optometry clinics across census metropolitan areas (CMAs) (main map); inset map of eastern provinces (**a**) inset map of Southern Ontario (**b**), and inset map of British Columbia (**c**). Note: Census subdivisions (CSDs) are classified into different metropolitan influenced zones (MIZs) across Canadian provinces. Labels: BC=British Columbia; AB = Alberta; SK=Saskatchewan; MB = Manitoba; ON=Ontario; QC = Quebec; NB=New Brunswick; PE = Prince Edward Island; NS=Nova Scotia; NL = Newfoundland and Labrador; YT = Yukon Territory; NT = Northwest Territory; NT = Nunavut Territory. ESRI ArcMAP 10.6 was used to prepare the maps (University of Saskatchewan license). The development ofbackground layers of: health region boundaries, water bodies, city locations, and provincial boundaries are from Statistics Canada’s Geography (publicly available layers): https://www12.statcan.gc.ca/census-recensement/2011/geo/bound-limit/bound-limit-2016-eng.cfm

This analysis is based on 5959 optometrists working across Canada. As shown in Table 1, we generated geographic locations using the following geocoding methods: postal code geocoding (*n* = 5835; 97.9%), and Google Maps (*n* = 114; 1.9%). There were 10 optometrists (0.17%) where address information was not provided, and these were excluded from the analysis. Figure 2 presents the health region level distribution of optometrists by relative rurality (based on MIZ). Out of the total sample of optometrists, 4750 (i.e., 79.7%) were located within urban census metropolitan or agglomeration areas (CMAs; CAs, brown color). The remainder of optometrists were distributed within different MIZs: 668 (i.e., 11.2%) within strong MIZs; 103 (1.7%) within moderate MIZs; 427 (7.2%) within weak/no MIZs. Optometrist provincial counts across different MIZs are shown in Additional file 1: Appendix 1.

**Table 1:** Total population with number of health regions across optometrist ratios using standard deviation (SD) classification scheme [population (HR count)]

Canadian Provinces (from west to east)	Population numbers (and count of health regions) arcos Optometrist Ratio (per 10,000 population) categorizes					Total Population (HR count)
	*Low [< −1.5 SD]*	*Moderately low [− 1.5 SD to − 0.50 SD]*	*Moderate [− 0.5 SD to + 0.50 SD]*	*Moderately high [+ 0.5 SD to + 1.5 SD]*	*High [> + 1.5 SD]*	
British Columbia		582,563 (4)	3,020,551 (8)	965,085 (3)	79,856 (1)	4,648,055 (16)
Alberta		903,389 (2)	291,112 (1)	2,872,674 (2)		4,067,175 (5)
Saskatchewan	35,453 (3)	151,741 (3)	288,419 (5)	622,739 (2)		1,098,352 (13)
Manitoba	199,821 (2)	192,061 (1)	886,483 (2)			1,278,365 (5)
Ontario		512,148 (4)	8,191,001 (20)	4,133,395 (10)	611,950 (2)	13,448,494 (36)
Quebec	30,329 (2)		2,328,266 (7)	5,216,366 (8)	589,400 (1)	8,164,361 (18)
New Brunswick		239,348 (3)	209,256 (1)	298,497 (3)		747,101 (7)
Nova Scotia			923,598 (4)			923,598 (4)
Prince Edward Island			142,907 (1)			142,907 (1)
Newfoundland and Labrador		390,954 (2)	128,762 (2)			519,716 (4)
**Canada**	265,603 (7)	2,972,204 (19)	16,410,355 (51)	14,108,756 (28)	1,281,206 (4)	35,038,124 (109)

*SD* standard deviation, *HR* health regions

**Fig. 3:**
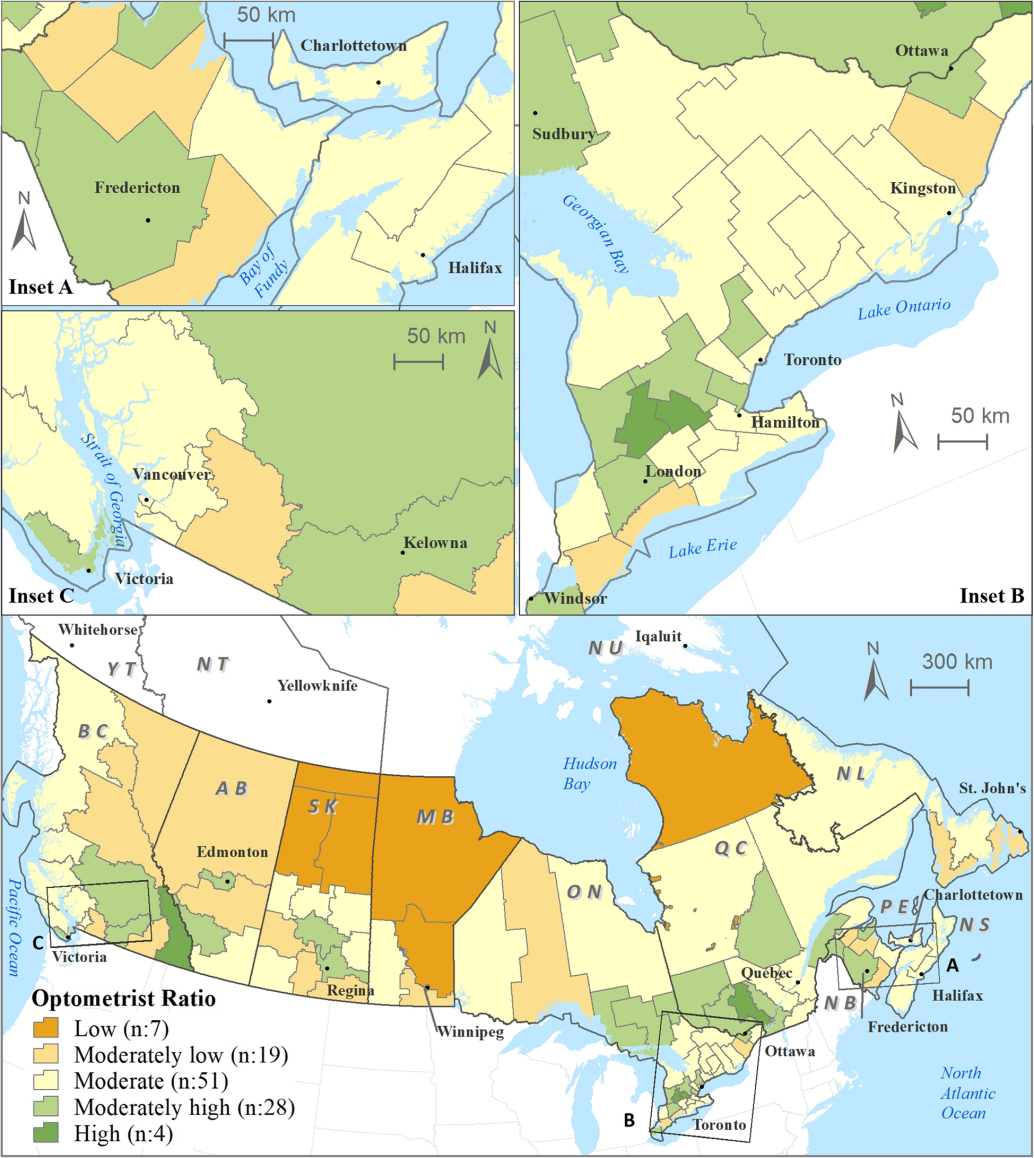
Distribution of optometrist ratio across Canadian health regions (main map); inset map of eastern provinces (**a**) inset map of Southern Ontario (**b**), and inset map of British Columbia (**c**). Note: Optometrist ratio is in the form of number of optometrists per 10,000 population and ‘n’ represents the count of health regions. Labels: BC=British Columbia; AB = Alberta; SK=Saskatchewan; MB = Manitoba; ON=Ontario; QC = Quebec; NB=New Brunswick; PE = Prince Edward Island; NS=Nova Scotia; NL = Newfoundland and Labrador; YT = Yukon Territory; NT = Northwest Territory; NT = Nunavut Territory. ESRI ArcMAP 10.6 was used to prepare the maps (University of Saskatchewan license). The development ofbackground layers of: health region boundaries, water bodies, city locations, and provincial boundaries are from Statistics Canada’s Geography (publicly available layers): https://www12.statcan.gc.ca/census-recensement/2011/geo/bound-limit/bound-limit-2016-eng.cfm

The average distribution of optometrists across 109 Canadian health regions was 1.70 optometrists per 10,000 people (range = 0.13 to 2.92) whereas the average proportion of eye care utilization by health regions was 41.87% (range = 32.11 to 51.77%; SD = 4.01). Figure 3 shows the distribution of optometrists HRs. Regarding optometrist ratio per 10,000 people: out of 109 HRs, 26 were in the lower categories, 51 HRs in the moderate, and 32 HRs in the higher categories as seen in the sum of values given in the legend of Fig. 3. Table 1 presents the province wide distribution of the total population (and HR counts) across the optometrist ratio categories (5-classes based on the standard deviation approach). About 9.2% of total population (i.e., 3.24 million) in 26 HRs from eight provinces (i.e. all except NS and PE) fall under the lower categories of optometrists per population ratios (i.e., < − 1.5 SD; − 1.5 SD to − 0.50 SD).

**Fig. 4:**
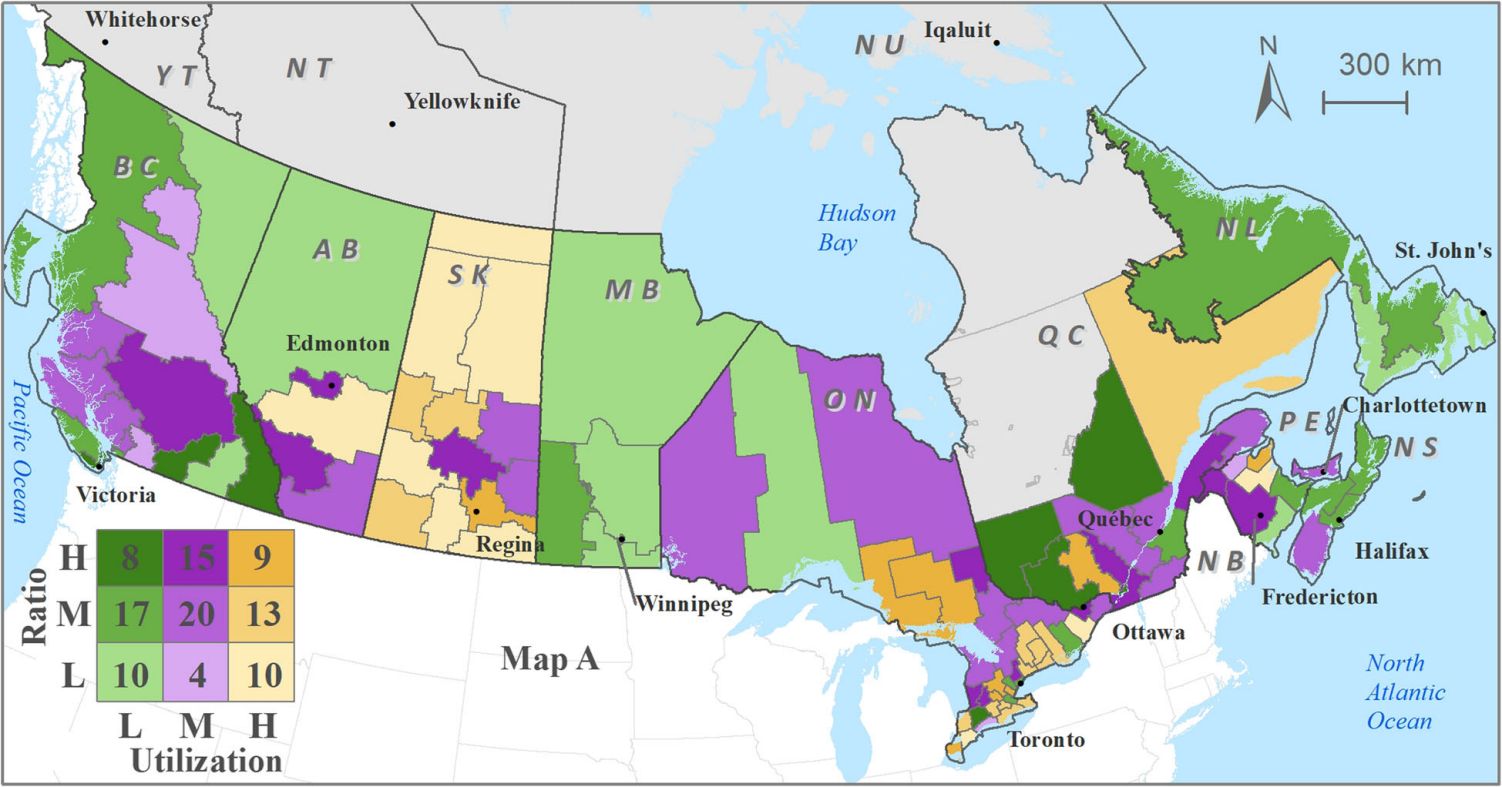
Cross-classification map of optometrist per 10,000 population (Ratio) with self-reported utilization of eye services; Labels: BC=British Columbia; AB = Alberta; SK=Saskatchewan; MB = Manitoba; ON=Ontario; QC = Quebec; NB=New Brunswick; PE = Prince Edward Island; NS=Nova Scotia; NL = Newfoundland and Labrador; YT = Yukon Territory; NT = Northwest Territory; NT = Nunavut Territory. L = low; M = moderate; H = high. ESRI ArcMAP 10.6 was used to prepare the maps (University of Saskatchewan license). The development ofbackground layers of: health region boundaries, water bodies, city locations, and provincial boundaries are from Statistics Canada’s Geography (publicly available layers): https://www12.statcan.gc.ca/census-recensement/2011/geo/bound-limit/bound-limit-2016-eng.cfm

**Fig. 5:**
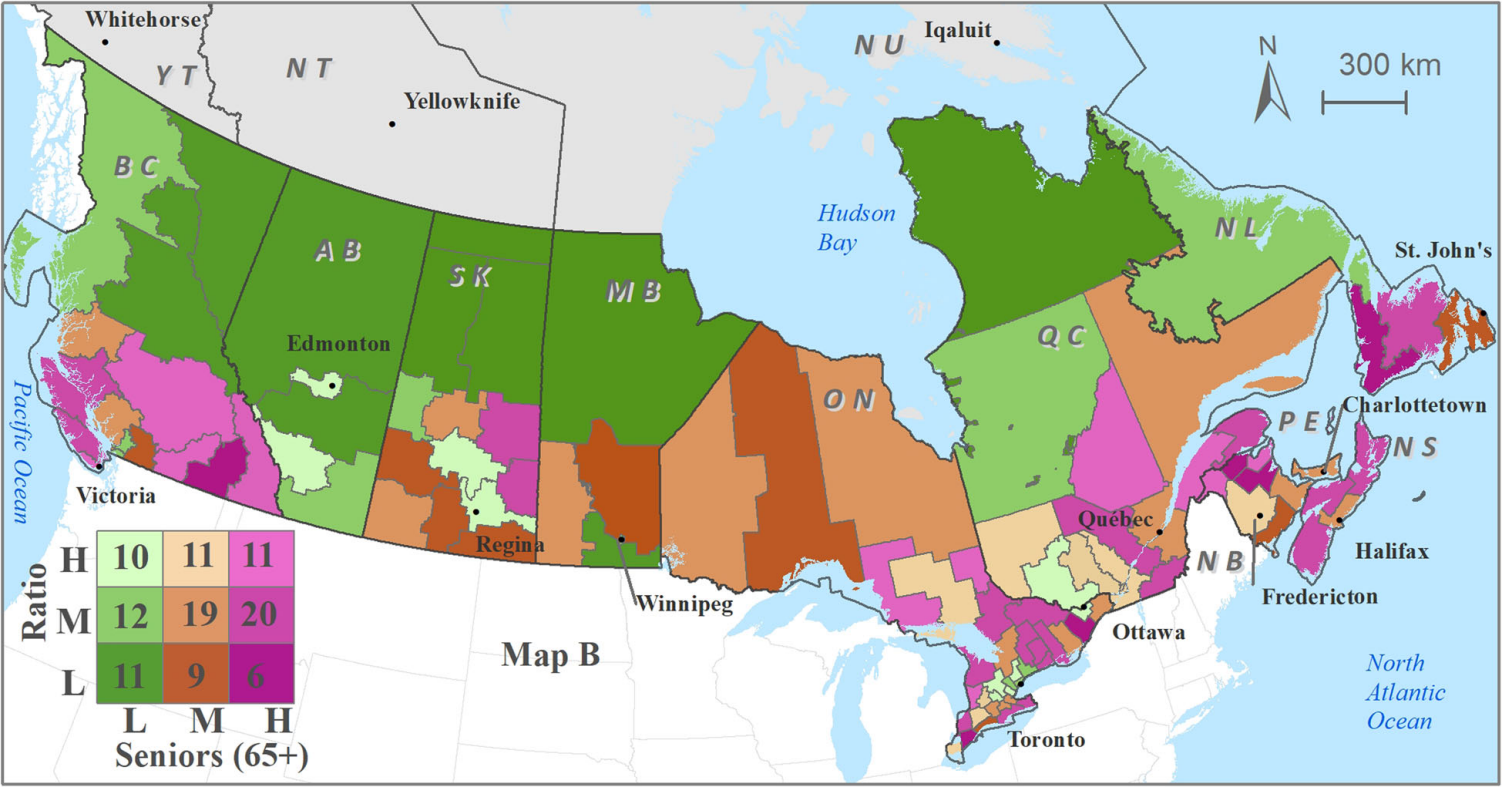
Cross-classification map of optometrist per 10,000 population (Ratio) with seniors population; Labels: L = low; M = moderate; H = high. ESRI ArcMAP 10.6 was used to prepare the maps (University of Saskatchewan license). The development ofbackground layers of: health region boundaries, water bodies, city locations, and provincial boundaries are from Statistics Canada’s Geography (publicly available layers): https://www12.statcan.gc.ca/census-recensement/2011/geo/bound-limit/bound-limit-2016-eng.cfm

**Fig. 6:**
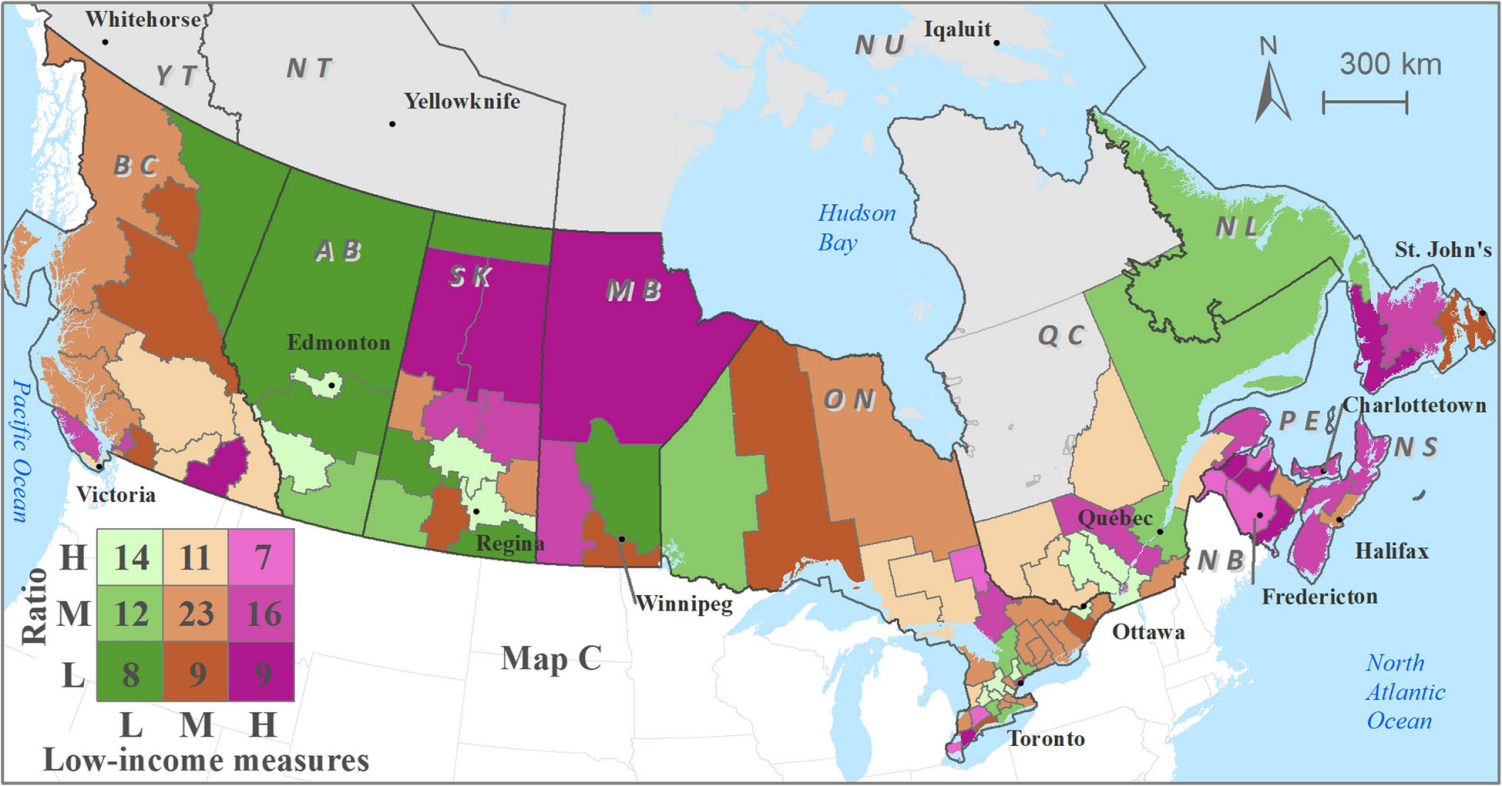
Cross-classification map of optometrist per 10,000 population (Ratio) with low-income families; Labels: L = low; M = moderate; H = high. ESRI ArcMAP 10.6 was used to prepare the maps (University of Saskatchewan license). The development ofbackground layers of: health region boundaries, water bodies, city locations, and provincial boundaries are from Statistics Canada’s Geography (publicly available layers): https://www12.statcan.gc.ca/census-recensement/2011/geo/bound-limit/bound-limit-2016-eng.cfm

**Fig. 7:**
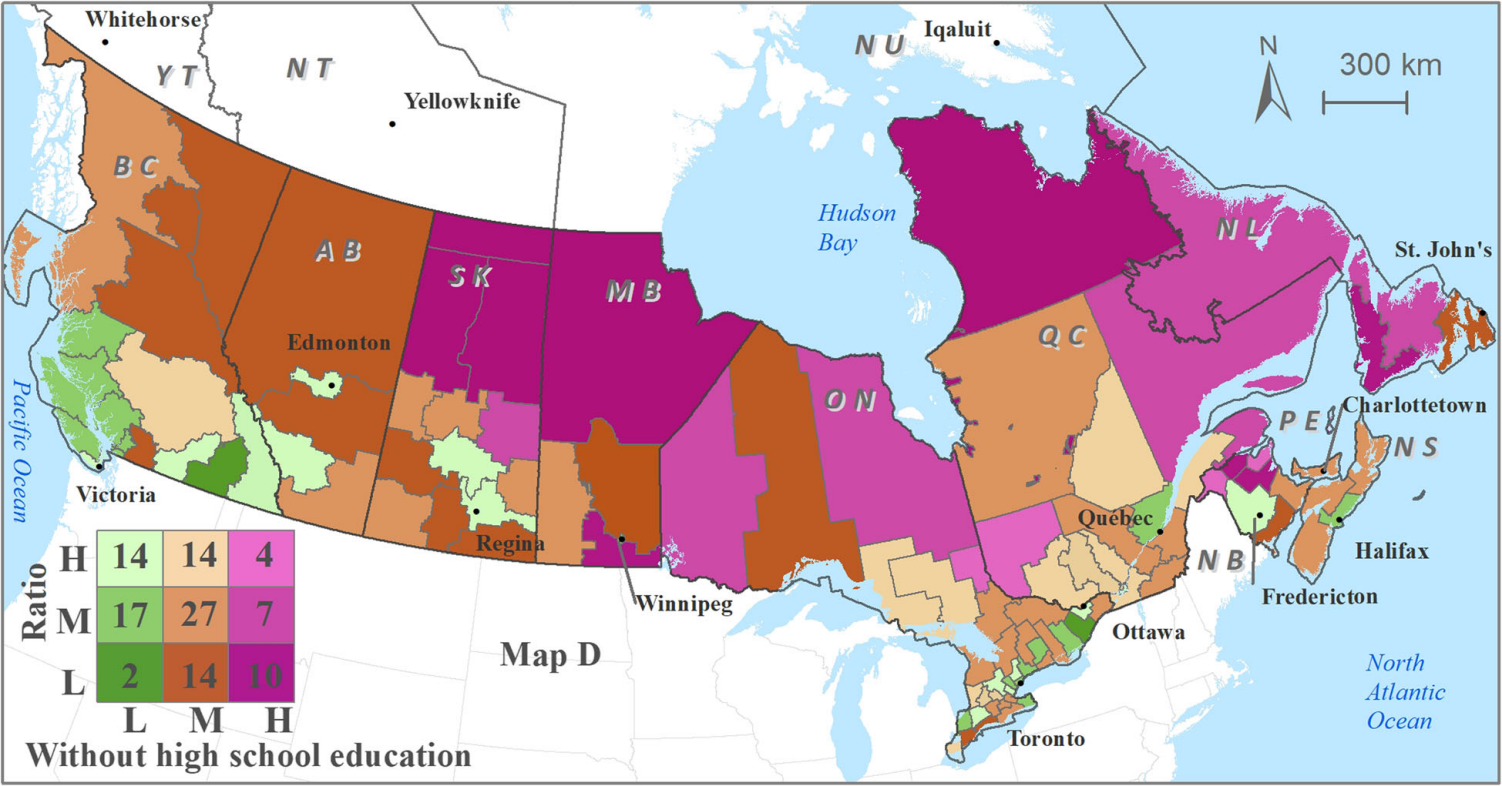
Cross-classification map of optometrist per 10,000 population (Ratio) with population without high school education; Labels: L = low; M = moderate; H = high. ESRI ArcMAP 10.6 was used to prepare the maps (University of Saskatchewan license). The development ofbackground layers of: health region boundaries, water bodies, city locations, and provincial boundaries are from Statistics Canada’s Geography (publicly available layers): https://www12.statcan.gc.ca/census-recensement/2011/geo/bound-limit/bound-limit-2016-eng.cfm

Optometrist distribution patterns relative to utilization of eye care services demonstrates moderate-low optometrist availability in health regions with moderate-high utilization of eye care services (Fig. 4) and in health regions with relatively higher percentage of population subgroups (higher than the national average) (Figs. 5, 6 and 7). Cross-classification between the ratio and use variables where 106 HRs were divided into nine category combinations (Fig. 4). Health regions with low optometrist ratio values relative to the low utilization of eye care are located throughout Canada. There are nine HRs (8.5%) that fell within the high utilization-high distribution ratio combination (1 HR from each of NB, QC, and SK, 6 from ON). Ten HRs (9.4%) from the following provinces were found to have a low utilization-low distribution ratio combination: 1 HR from each of NB, ON, and AB, 2 from each of BC, and NL, 3 from MB. Ten HRs (9.4%) fell within high utilization-low distribution ratio combination: 1 HR from each of NB, and AB, 2 from ON, 3 from SK). Eight HRs (7.5%) had a low utilization-high distribution ratio combination: 1 HR from ON, 3 from BC, and 4 from QC. Various other combinations with moderate utilization or distribution were found in 69 HRs distributed across all 10 provinces.

Cross-classification between the ratios and socio-demographic characteristics (3 variables) were divided into nine category combinations are shown in Fig. 5, 6 and 7. Health regions with low optometrist ratios relative to the high percent of sociodemographic ‘need’ characteristics (Fig. 5, 6 and 7) are located throughout Canada. For example, in the case of seniors’ population (Fig. 5), 11 HRs (8.5%) fell within the high percent of seniors’ population-high optometrist distribution ratio combination (2 HRs from each of NB, and QC, 3 from ON, 4 from BC). In the case of a low percent of seniors’ population -low optometrist distribution ratio combination, 11 health regions (9.4%) from the following provinces were found: 2 HRs from each of QC, MB, AB, and BC, and 3 from SK. High percent of seniors’ population-low optometrist distribution ratio values were found in 6 HRs (9.4%; 1 HR from each of NL, and BC, 2 from NB, and ON). Low percent of seniors’ population-high optometrist distribution ratio values were found in 10 HRs (7.5%; 1 HR from QC, 2 from each of SK, and AB, 5 from ON). Various other combinations with moderate percent of seniors’ population or distribution were found in 71 HRs.

## Discussion

This research provides an important overview of distribution patterns of vision care services provided by optometrists across Canadian health regions. Specifically, this work has identified variation and poorly-served health regions to optometry services across ten Canadian provinces in relation to self-reported use of vision care services, and census-based socio-demographic characteristics (i.e. seniors, lower income and education levels). Overall, there is an uneven distribution of optometrists across Canadian health regions. Across Canada, most optometrists are concentrated in densely populated areas of Calgary, Edmonton, Saskatoon, Regina, Southwestern Ontario (London), Central Ontario (except Toronto), Ottawa, Montreal, and Fredericton. Canada’s most populous urban centres, Vancouver and Toronto, have moderate optometrist ratios (Fig. 3). A few moderate-high optometrist concentration areas are located in Kelowna and Kootenay regions in British Columbia, Waterloo and Thunder Bay areas in Ontario, areas in and around Gatineau and Mon-Tremblant, Riviere-du-Loup in Quebec, Edmundston in New Brunswick (Fig. 3). The majority of optometrists (*n* = 4750 or 79.7%) were located within urban centers consistent with distribution of physicians, nurses and physiotherapists [36].

The results of this study indicate health regions with low optometrist ratio values relative to the low utilization of eye care and relatively high percent of sociodemographic ‘need’ characteristic which are located throughout Canada. Our results align with what was found by Khan, Trope, Wedge [37] in Prince Edward Island where low vision care utilisation is attributed to barriers in accessing government-insured ophthalmologists. In previous studies on eye care services/utilization, different socio-demographic characteristics have been found to be related to distribution of optometry services. A further analysis of the Canadian Longitudinal Study on Aging [24] identified disparities in the utilization of eye care services in Canada with less educated populations (less than bachelor’s degree) and those with lower income less likely to use these services. For example, in an analysis of the Canadian Longitudinal Study on Aging, Aljied, Aubin, Buhrmann [38] reported that older age and income are critical factors for visual impairment. A study in Newfoundland and Labrador also found that low socioeconomic status (including lack of government insurance) and living in non-urbanized areas are related to the under-utilization of eye care providers [26].

Adequate geographical distribution of health care services is an important contributor to equitable access to health care [39]. The actual use of services is called realized access [40]. Potential access, on the other hand, relates to an individual’s perception of their ability to access needed care which may be influenced by other factors, such as geographical distribution of services [40]. Our findings show uneven geographical distribution of optometrists relative to utilization and key socio-demographic indicators across Canadian health regions, suggesting that there are likely gaps in both potential and realized equitable access to care. It should be noted that data regarding optometrist-use do not directly relate to potential population health need because those who need the service may not necessarily be able to access care for a variety of reasons. Thus, potential access and use (i.e. realized access) are not synonymous because an individual may need to overcome barriers, such as geographical location and travel barriers, that limit her or his access to a particular service in order to use it [41]. Additionally, the perceived health care needs of an individual do not necessarily reflect use of or access to particular services [42]. This research focused on population level distribution of need variables, rather than individual or health outcome characteristics. Further research should examine whether individual-level characteristics such as age, income, education, and other relevant contextual variables are associated with use of eye care services, as has been done in research examining vision care providers in the United States among adults with diabetes [24].

There are several contextual factors in relation to vision care services that this research does not examine, such as funding policies and insurance availability [43]. It is important to note that this research only considered geographical barriers and not cost barriers. Population groups such as seniors, those with low incomes or educational attainment may be vulnerable to both cost and geographical barriers in health seeking. In Canada, there is variable publicly-funded coverage of optometry services [20, 44] and not all residents may have additional private health insurance to offset the costs [45]. In provinces without government-insured optometric services, the pattern of utilization may reflect mixed barriers from both geography and finance.

### Limitations and other considerations

Interpreting the findings of this report should be viewed in light of limitations in data quality pertaining to optometrist location, utilization and other geographical factors. The optometrist ratios are most likely overestimated due to optometrist counts used for analysis instead of full-time equivalents. Optometrist practice locations are based on the postal code that may have some geographic uncertainty resulting in a potential misalignment with health regions. Self-reported utilization of vision care services is likely to be overestimated as both optometrist and ophthalmologist providers are reported together as use of vision care services in the CCHS and cannot be separated. A notable consideration between accessing ophthalmologists and optometrists in Canada, is that optometrists are direct access providers, meaning that no referral is required, whereas accessing an ophthalmologist requires a referral from a primary care provider such as a family physician. In addition, the CCHS only surveyed respondents aged 12 an over, thus utilization of eye care services in those less than 12 years of age will not be captured. In some cases, the CCHS data is available for groups of neighboring health regions only (primarily to increase the sample size). As of April 1, 2015, Nova Scotia has one Provincial Health Authority, with four management zones. These management zones are an aggregation of the nine former health authorities. A further limitation is related to data used in this study were derived from different periods (i.e., Optometrist distribution from 2018, the CCHS utilization data from 2013 to 2014, and the census data from 2016) that can affect the apparent association between the optometrist ratio and other variables. Since the number of optometrists in 2013–14, the same year when utilization data was measured, would certainly be lower than those in 2018 and census data in 2013–14 might be slightly different from those in 2016 where some elderly might be deceased.

## Conclusion

This research provides an overview of distribution patterns of vision care services provided by optometrists across Canadian health regions. The results show optometrists are located across Canadian health regions in a variety of rural and urban settings, with greater concentration in urban settings. However, there is considerable variation with a number of poorly-served health regions by optometry services identified across ten Canadian provinces relative to both self-reported use of vision care services, and census-based distribution of socio-demographic characteristics (i.e. seniors, lower income and education levels). This research identifies potential gaps in geographic access regarding “supply” of optometrist vision care services relative to socio-demographic “need” factors. These findings provide a better understanding of accessibility to eye care services provided by optometrists and can be used to inform workforce and service delivery policies and planning at national, provincial, and health region levels.

## Supplementary information

**Additional file 1: Appendix 1.** Provincial summary of optometrist count and geocoding techniques used for mapping practice sites. **Appendix 2.** Summary: Optometrist count by province and Statistical Area Classification (SAC).

## Data Availability

The data that support the findings of this study are available from Statistics Canada (Government of Canada). Thee CCHS related data can be download from the ODESI website (Ontario Data Documentation, Extraction Service and Infrastructure; https://search1.odesi.ca/#/).

## Abbreviations

CA: Census agglomeration
CAO: Canadian association of optometrists
CHP: Contacts with health professionals
CMA: Census metropolitan areas
CCHS: Canadian community health survey
CSD: Census subdivisions
CT: Census tract
DA: Dissimilation areas
DB: Dissemination block
FTE: Full-time-equivalent
GIS: Geographical Information Systems
HA: Health authority
HR: Health regions
HU: Health unit
MEP: Multiple enhanced postal codes
MIZ: Metropolitan influence zone
PHC: Primary health care
RSTs: Rural and small towns
RHA: Regional health authority
SES: Socioeconomic status
SAC: Statistical area classification
SD: Standard deviation

## References

[1] Health Canada. About Primary Health Care Ottawa2006 [cited 2012 16 July]. Available from: http://www.hc-sc.gc.ca/hcs-sss/prim/about-apropos-eng.php#a1.

[2] DiCenso A, Auffrey L, Bryant-Lukosius D, Donald F, Martin-Misener R, Matthews S (2007). Primary health care nurse practitioners in Canada. Contemp Nurse.

[3] Hutchison B, Levesque J-F, Strumpf E, Coyle N (2011). Primary health care in Canada: systems in motion. Milbank Quarterly.

[4] The Conference Board of Canada. Improving primary health care through collaboration: Briefing 1 - current knowledge about interprofessional teams in Canada. Ottawa, ON: The Conference Board of Canada, 2012 Contract No.: 13–145.

[5] Reid GJ, Freeman TR, Thind A, Stewart M, Brown JB, Vingilis ER (2009). Access to family physicians in southwestern Ontario. Healthcare Policy.

[6] Shah TI, Clark AF, Seabrook JA, Sibbald S, Gilliland JA. Geographic accessibility to primary care providers: Comparing rural and urban areas in Southwestern Ontario. The Canadian Geographer / Le Géographe canadien 2019;0(0).

[7] Khan AA (1992). An integrated approach to measuring potential spatial access to health care services. Socio Econ Plan Sci.

[8] Luo W, Wang F (2003). Measures of spatial accessibility to health care in a GIS environment: synthesis and a case study in the Chicago region. Environ Planning B: Planning Design.

[9] Bissonnette L, Wilson K, Bell S, Shah T (2012). Neighbourhoods and potential access to health care: the role of spatial and aspatial factors. Health Place.

[10] Guagliardo MF, Ronzio CR, Cheung I, Chacko E, Joseph JG (2004). Physician accessibility: an urban case study of pediatric providers. Health Place.

[11] Ngui A, Apparicio P. Optimizing the two-step floating catchment area method for measuring spatial accessibility to medical clinics in Montreal. BMC Health Serv Res. 2011;11. 10.1186/1472-6963-11-166 PMC3142205 21745402

[12] Roeger LS, Reed RL, Smith BP (2010). Equity of access in the spatial distribution of GPs within an Australian metropolitan city. Australian J Primary Health.

[13] Bell S, Wilson K, Bissonette L, Shah T (2013). Access to primary health care: does neighbourhood of residence matter?. Ann Assoc Am Geogr.

[14] Bell S, Wilson K, Shah T, Gersher S, Elliott T (2012). Investigating impacts of positional error on potential health care accessibility. Spatial Spatio-temporal Epidemiol.

[15] Sanders LJ, Aguilar GD, Bacon CJ (2013). A spatial analysis of the geographic distribution of musculoskeletal and general practice healthcare clinics in Auckland, New Zealand. Appl Geogr.

[16] Crooks V, Schuurman N (2012). Interpreting the results of a modified gravity model: examining access to primary health care physicians in five Canadian provinces and territories. BMC Health Serv Res.

[17] Micieli JA (2014). Geographic distribution of ophthalmologists in Ontario: a 10-year review. Can J Ophthalmol.

[18] Bellan L, Buske L, Wang S, Buys YM (2013). The landscape of ophthalmologists in Canada: present and future. Can J Ophthalmol.

[19] Al Ali A, Hallingham S, Buys YM (2015). Workforce supply of eye care providers in Canada: optometrists, ophthalmologists, and subspecialty ophthalmologists. Can J Ophthalmol.

[20] Hong CJ, Trope GE, Buys YM, Robinson BE, Jin Y-P (2014). Does government assistance improve utilization of eye care services by low-income individuals?. Can J Ophthalmol.

[21] Jin Y-P, Trope GE (2011). Eye care utilization in Canada: disparity in the publicly funded health care system. Can J Ophthalmol.

[22] Perruccio AV, Badley EM, Trope GE (2010). A Canadian population-based study of vision problems: assessing the significance of socioeconomic status. Can J Ophthalmol.

[23] Xu K, Trope GE, Buncic R, Jin Y-P (2012). Utilization of eye care providers by Canadian adolescents: evidence from the Canadian community health survey. Can J Ophthalmol.

[24] Chou C-F, Zhang X, Crews JE, Barker LE, Lee PP, Saaddine JB (2012). Impact of geographic density of eye care professionals on eye care among adults with diabetes. Ophthalmic Epidemiol.

[25] Lee CS, Su GL, Baughman DM, Wu Y, Lee AY (2017). Disparities in delivery of ophthalmic care; an exploration of public Medicare data. PLoS One.

[26] Lee EY, Cui K, Trope GE, Buys YM, Chan CH, Thavorn K (2018). Eye care utilisation in Newfoundland and Labrador: access barriers and vision health outcomes. Can J Ophthalmol.

[27] Alter DA, Stukel T, Chong A, Henry D (2011). Lesson from Canada’s universal care: socially disadvantaged patients use more health services. Still Have Poorer Health Health Affairs.

[28] Aljied R, Aubin M-J, Buhrmann R, Sabeti S, Freeman EE (2018). Eye care utilization and its determinants in Canada. Can J Ophthalmol.

[29] Statcan. Canadian Community Health Survey - Annual Component (CCHS) Ottawa, ON: Statistics Canada; 2020 [updated 2020-01-02; cited 2020 Feb 15]. Available from: https://www23.statcan.gc.ca/imdb/p2SV.pl? Function=getSurvey&SDDS=3226.

[30] Aldridge H (2017). How do we measure poverty?: Maytree Foundation.

[31] Lammam C, MacIntyre H (2016). An introduction to the state of poverty in Canada: Fraser Institute.

[32] Statcan. Census metropolitan influenced zone (MIZ): Statistics Canada; 2015 [cited 2017 June 03]. Available from: http://www12.statcan.gc.ca/census-recensement/2011/ref/dict/geo010-eng.cfm.

[33] Brewer CA, Pickle L (2002). Evaluation of methods for classifying epidemiological data on choropleth maps in series. Ann Assoc Am Geogr.

[34] James WL, Cossman RE, Cossman JS, Campbell C, Blanchard T (2004). A brief visual primer for the mapping of mortality trend data. Int J Health Geogr.

[35] GIS Library. Geospatial data Saskatoon, SK: University of Saskatchewan; 2014 [cited 2014 March 14]. Available from: http://library.usask.ca/murray/data-and-gis/GIS.php.

[36] Shah TI, Milosavljevic S, Trask C, Bath B. Mapping Physiotherapy Utilization in Canada in Relation to Physiotherapist Distribution. Physiotherapy Canada 2019. 10.3138/ptc-2018-0023 PMC6830420 31719717

[37] Khan AM, Trope GE, Wedge R, Buys YM, El-Defrawy S, Chen Q (2018). Policy implications of regional variations in eye disease detection and treatment on Prince Edward Island: a repeated cross-sectional analysis, 2010–2012. BMC Health Serv Res.

[38] Aljied R, Aubin M-J, Buhrmann R, Sabeti S, Freeman EE (2018). Prevalence and determinants of visual impairment in Canada: cross-sectional data from the Canadian longitudinal study on aging. Can J Ophthalmol.

[39] Campbell J, Buchan J, Cometto G, David B, Dussault G, Fogstad H (2013). Human resources for health and universal health coverage: fostering equity and effective coverage. Bull World Health Organ.

[40] Aday LA, Andersen RM (1981). Equity of access to medical care: a conceptual and empirical overview. Med Care.

[41] Thorpe JM, Thorpe CT, Kennelty KA, Pandhi N (2011). Patterns of perceived barriers to medical care in older adults: a latent class analysis. BMC Health Serv Res.

[42] Allin S, Grignon M, Le Grand J (2010). Subjective unmet need and utilization of health care services in Canada: what are the equity implications?. Soc Sci Med.

[43] Zhang X, Andersen R, Saaddine JB, Beckles GLA, Duenas MR, Lee PP (2008). Measuring access to eye care: a public health perspective. Ophthalmic Epidemiol.

[44] Chan CH, Trope GE, Badley EM, Buys YM, Jin Y-P (2014). The impact of lack of government-insured routine eye examinations on the incidence of self-reported Glaucoma, cataracts, and vision loss. Invest Ophthalmol Vis Sci.

[45] SANOFI. WINDS OF CHANGE: new directions in employee health benefits. Laval, QC: 2017.

