# Supplementary material for: Geographic availability to optometry services across Canada: mapping distribution, need and self-reported use
Source: BMC Health Serv Res. 2020 Jul 10;20:639. doi: 10.1186/s12913-020-05499-6 (PMC7350740; doi:10.1186/s12913-020-05499-6)
Supplement: Supplementary file 1 — Additional file 1: Appendix 1. Provincial summary of optometrist count and geocoding techniques used for mapping practice sites. Appendix 2. Summary: Optometrist count by province and Statistical Area Classification (SAC). [file 12913_2020_5499_MOESM1_ESM.doc]

**Appendix 1.** Provincial summary of optometrist count and geocoding techniques used for mapping practice sites

| Province | Data Source | Optometrist count | | Geocoding Technique | | |
| --- | --- | --- | --- | --- | --- | --- |
|  |  | Raw* | Refined* | EP Point | Google/ manual | Not located |
| British Columbia | College of Optometrists of BC | 725 | 730 | 715 | 15 |  |
| Alberta | Alberta College of Optometrists | 749 | 742 | 732 | 10 |  |
| Saskatchewan | Provincial College | 176 | 178 | 177 | 1 |  |
| Manitoba | Provincial College | 173 | 174 | 165 |  | 9 |
| Ontario | Provincial College | 2,349 | 2,355 | 2,280 | 75 |  |
| Quebec | Provincial College | 1,447 | 1,443 | 1,432 | 10 | 1 |
| New Brunswick | Provincial College | 118 | 118 | 117 | 1 |  |
| Nova Scotia | Provincial College | 132 | 131 | 130 | 1 |  |
| Prince Edward Island | PEI College of Optometrists | 21 | 21 | 20 | 1 |  |
| Newfoundland and Labrador | Newfoundland & Labrador Association of Optometrists, and Newfoundland & Labrador College of Optometrists | 70 | 67 | 67 |  |  |
| Territories | n/a | 0 |  |  |  |  |
| Total (Canada) |  | 5,960 | 5,959 | 5,835 | 114 |  |

* presents optometrist count as per respect province; ** presents optometrist count within respective province based on the postal code (place of practice).

**Appendix 2.** Summary: Optometrist count by province and Statistical Area Classification (SAC)

| Province | Census metropolitan influenced zone | | | | | | | Total |
| --- | --- | --- | --- | --- | --- | --- | --- | --- |
|  | 1 | 2 | 3 | 4 | 5 | 6 | 7 |  |
| British Columbia | 509 | 69 | 106 | 9 | 13 | 23 |  | 729 |
| Alberta | 591 | 33 | 52 | 6 | 18 | 42 |  | 742 |
| Saskatchewan | 117 |  | 41 |  | 3 | 17 |  | 178 |
| Manitoba | 117 |  | 29 | 1 | 8 | 10 |  | 165 |
| Ontario | 1,996 | 50 | 171 | 45 | 59 | 35 |  | 2,356 |
| Quebec | 1,083 | 36 | 180 | 24 | 83 | 35 | 1 | 1,442 |
| New Brunswick | 41 | 22 | 20 | 6 | 12 | 16 |  | 117 |
| Nova Scotia | 56 |  | 31 | 11 | 14 | 21 |  | 133 |
| Prince Edward Island |  |  | 17 | 1 | 3 |  |  | 21 |
| Newfoundland and Labrador | 30 |  | 21 |  | 4 | 11 |  | 66 |
| Total (Canada) | 4,540 | 210 | 668 | 103 | 217 | 210 | 1 | 5,949 |

Note: 1 = all CSDs in census metropolitan areas (CMAs); 2= all CSDs in census agglomerations (CAs); 3= all CSDs in the provinces in the strong metropolitan influenced zone category; 4= all CSDs in the provinces in the moderate metropolitan influenced zone category; 5= all CSDs in the provinces in the weak metropolitan influenced zone category; 6= all CSDs in the provinces in the no metropolitan influenced zone category; 7= CSDs in the three territories (Yukon, Northwest Territories and Nunavut), except those that are components of a CA (currently the CAs of Whitehorse and Yellowknife).
